# Sulforaphane Modulates AQP8-Linked Redox Signalling in Leukemia Cells

**DOI:** 10.1155/2018/4125297

**Published:** 2018-11-18

**Authors:** Cecilia Prata, Carlotta Facchini, Emanuela Leoncini, Monia Lenzi, Tullia Maraldi, Cristina Angeloni, Laura Zambonin, Silvana Hrelia, Diana Fiorentini

**Affiliations:** ^1^Department of Pharmacy and Biotechnology, Alma Mater Studiorum, University of Bologna, Via Irnerio, 48-40126 Bologna, Italy; ^2^Department for Life Quality Studies, Alma Mater Studiorum, University of Bologna, Corso d'Augusto, 237-47921 Rimini, Italy; ^3^Department of Surgery, Medicine, Dentistry and Morphological Sciences, University of Modena and Reggio Emilia, Policlinico, Via del Pozzo, 71-41124 Modena, Italy; ^4^School of Pharmacy, University of Camerino, Via Gentile III da Varano, 62032 Camerino, Macerata, Italy

## Abstract

Sulforaphane, a biologically active isothiocyanate compound extracted from cruciferous vegetables, has been shown to exert cytotoxic effects on many human cancer cells, including leukemia. However, the exact molecular mechanisms behind the action of sulforaphane in hematological malignancies are still unclear. Like other cancer cells, leukemia cells produce high level of reactive oxygen species; in particular, hydrogen peroxide derived from Nox family is involved in various redox signal transduction pathways, promoting cell proliferation and survival. Recent evidence show that many tumour cell types express elevated level of aquaporin isoforms, and we previously demonstrated that aquaporin-8 acts as H_2_O_2_ transport facilitator across the plasma membrane of B1647 cells, a model of acute myeloid human leukemia. Thus, the control of AQP8-mediated H_2_O_2_ transport could be a novel strategy to regulate cell signalling and survival. To this purpose, we evaluated whether sulforaphane could somehow affect aquaporin-8-mediated H_2_O_2_ transport and/or Nox-mediated H_2_O_2_ production in B1647 cell line. Results indicated that sulforaphane inhibited both aquaporin-8 and Nox2 expression, thus decreasing B1647 cells viability. Moreover, the data obtained by coimmunoprecipitation technique demonstrated that these two proteins are linked to each other; thus, sulforaphane has an important role in modulating the downstream events triggered by the axis Nox2-aquaporin-8. Cell treatment with sulforaphane also reduced the expression of peroxiredoxin-1, which is increased in almost all acute myeloid leukemia subtypes. Interestingly, sulforaphane concentrations able to trigger these effects are achievable by dietary intake of cruciferous vegetables, confirming the importance of the beneficial effect of a diet rich in bioactive compounds.

## 1. Introduction

The consumption of whole plant foods as chemopreventive agents is highly recommended in the dietary guidelines on the basis of health benefits from dietary phytochemicals observed in epidemiological studies [1]. Among edible plants, cruciferous vegetables have been proved to exert potent anticarcinogenic effects owing to the presence of isothiocyanates, which are the hydrolytic products of glucosinolates. Among cruciferous vegetables, broccoli contains the highest concentration of the glucosinolate glucoraphanin, which is hydrolysed by myrosinase and gut microbiota, releasing sulforaphane, SFN (4-methylsulfinylbutyl isothiocyanate). In addition to its well-known anticancer activity [2], SFN has been demonstrated to possess cardioprotective [3], neuroprotective [4], and anti-inflammatory activities [5], suggesting a pleiotropic protective role for this nutraceutical compound.

The potent chemopreventive effect of SFN is based on its ability to target multiple mechanisms within the cell to control carcinogenesis. Many reports have shown that SFN prevents tumour initiation by both inhibiting phase I enzymes [6] and activating phase II detoxifying enzymes [7]. Moreover, SFN prevents uncontrolled cancer cell proliferation through the modulation of genes involved in apoptosis and cell cycle arrest [5, 8], angiogenesis [9, 10], and metastasis [11, 12].

SFN cytotoxic effects have also been demonstrated on hematological malignancies [13], and it has been reported that SFN treatment of HL-60 and acute lymphoblastic leukemia cells triggered apoptosis or cell cycle arrest [14–17]. Leukemia is one of the main cause of cancer-associated death, and the high susceptibility to treatment-related toxicity is still the major limit to the therapeutic success. Therefore, the identification and development of novel agents from natural products to counteract this disease are needed in order to maximize the therapeutic benefit and minimize antineoplastic drug resistance and treatment-related toxicity in patients treated with intensified doses of multiple drugs.

In the human erythromegakaryocytic cell line B1647, a model of acute myeloid leukemia, constitutively producing VEGF and expressing its tyrosine kinase receptor, VEGFR-2 [18], we demonstrated that VEGF signalling is coupled to NAD(P)H oxidase (Nox) activity [19]. In particular, H_2_O_2_ generated via Nox2- and Nox4-dependent pathways is involved in early signalling events, such as the maintenance of the VEGFR-2 phosphorylation state, and also in the modulation of downstream events leading to cell proliferation and survival [20, 21]. It has to be pointed out that H_2_O_2_-derived Nox is formed outside the cell and have to cross the membrane to reach its cytosolic targets. To this regard, it has been reported that specific aquaporin isoforms are capable of funneling H_2_O_2_ across the plasma membrane in many cell types [22, 23]. In particular, AQP8 isoform has demonstrated the ability to channel H_2_O_2_ through the plasma membrane in B1647 cell line [24, 25], HeLa [26], and B [27] cells.

Furthermore, tumour cells overexpress AQPs, and a positive correlation exists between histological tumour grade and the AQP expression as compared to normal tissues [28–30].

The inhibition of AQP8-mediated H_2_O_2_ entry into the cell, or the decreased AQP8 expression, entails that Nox-derived H_2_O_2_ cannot exert its growth-promoting effects. Therefore, the control of AQP8-mediated H_2_O_2_ transport provides a novel mechanism to regulate cell signalling and survival.

This study aimed at evaluating the potential anticancer activity of SFN in B1647 leukemia cell line, focusing on AQP8 function and expression. We also investigated the effect of SFN on Nox2, Nox4, and peroxiredoxin expression and on the phosphorylation state of VEGFR-2 and Akt.

## 2. Materials and Methods

### 2.1. Chemicals and Reagents

Dulbecco's modified Eagle's medium (DMEM), Roswell Park Memorial Institute (RPMI) 1640 medium, penicillin, streptomycin, L-glutamine, phytohaemagglutinin (PHA), 3-(4,5-dimethylthiazol-2-yl)-2,5-diphenyltetrazolium bromide (MTT), DAPI, RIPA lysis buffer, 10% SDS solution, mammalian protease inhibitor mixture, phosphatase inhibitor cocktail PhosSTOP (Roche), Laemmli sample buffer containing 2-mercaptoethanol, Tris-HCl, bovine serum albumin (BSA), Hank's Balanced Salt Solution (HBSS), 2,7-dichlorodihydrofluorescin diacetate (DCFH-DA), plumbagin from *Plumbago indica* (5-hydroxy-2-methyl-1,4-naphthoquinone), and all other chemicals were purchased from Sigma-Aldrich. Iscove's Modified Dulbecco's medium (IMDM) with L-glutamine, foetal bovine serum (FBS), and human serum AB male (HS) were purchased from Biowest. DL-Sulforaphane (SFN) (LKT Laboratories) was dissolved in DMSO and stored at −20°C at a stock concentration of 10 mM. Absolute RNA Miniprep Kit was from Agilent Technologies; RNA-to-cDNA conversion kit was from Applied Biosystems; SsoAdvanced™ Universal SYBR Green Supermix was from Bio-Rad Laboratories; and RT-PCR primers for AQP8, *β*-2-microglobulin, and actin were manufactured from Sigma-Aldrich. SiRNA against Nox4 and scrambled were obtained from Ambion by Life Technologies (USA). Mini-PROTEAN® TGX™ precast gels 4–20%, Precision Plus Protein™ Unstained Standards, Clarity™ Western ECL Substrate, and DC™ protein assay were from Bio-Rad Laboratories. Nitrocellulose membranes were from GE Healthcare. Primary antibodies against: AQP8 (#WH0000343) and *β*-actin (#A5441) were from Sigma-Aldrich, phospho-Akt (Ser473) (#4058) and Prx-1 (#8499) from Cell Signalling Technologies, phospho-VEGFR-2 from Thermo Scientific, gp91-phox (Nox2) from Millipore, and Nox4 from Santa-Cruz. Secondary antibodies: horseradish peroxidase-conjugated secondary antibodies anti-rabbit (#7074) and anti-mouse (#7076) were purchased from Cell Signalling Technologies; goat anti-Mouse IgG (H + L) Highly Cross-Adsorbed Secondary Antibody, Alexa Fluor® Plus 488 (#A32723) was from Thermo Scientific.

### 2.2. Cell Culture

B1647 erythromegakaryocytic cell line, established from the bone marrow of a patient with acute myelogenous leukaemia (AML), is cultured in IMDM supplemented with 5% (*v*/*v*) heat inactivated HS, L-glutamine, 100 U/mL penicillin, and 100 *μ*g/mL streptomycin in a humidified incubator maintained at 37°C and 5% CO_2_.

Human fibroblasts were grown and kindly provided by Professor A. Lorenzini, University of Bologna.

Peripheral blood lymphocytes (PBL) were isolated by density gradient centrifugation with Histopaque-1077 from whole peripheral blood of healthy donors. PBL were cultured at 37°C and 5% CO_2_ in RPMI-1640 supplemented with 1% penicillin/streptomycin, 15% heat inactivated FBS, 1% L-glutamine, and 0.5% PHA.

### 2.3. Cell Viability

Cell viability was evaluated by the MTT assay. Cells were treated with increasing concentrations of SFN (5, 10, or 30 *μ*M) for 24 h in 96-well plates, then incubated with 0.5 mg/mL MTT for 4 h at 37°C. The blue-violet formazan salt crystals formed were dissolved with a solubilisation solution (10% SDS, 0.01 M HCl) keeping the plates overnight at 37°C and 5% CO_2_ in a humidified atmosphere. The absorbance at 570 nm was measured using a multiwell plate reader (Wallac Victor^2^, PerkinElmer).

### 2.4. Analysis of mRNA Expression by RT-PCR

After 24 h treatment with SFN (1, 5, or 10 *μ*M), total RNA was extracted from B1647 cells using Absolutely RNA Miniprep Kit according to the manufacturer's recommendations. RNA quantification was performed using a NanoVue spectrophotometer (GE Healthcare). mRNA was reverse-transcribed into cDNA starting from 1 *μ*g of total RNA using a high capacity RNA-to-cDNA Conversion Kit. PCR was carried out in a total volume of 20 *μ*L containing 2 *μ*L of cDNA, 10 *μ*L SsoAdvanced™ Universal SYBR Green Supermix, and 1 *μ*L (500 nM) of each primer. The specific primers used were produced by Sigma-Aldrich: AQP8 (forward sequence: TTCTCCATCGGCTTTGCCGTCA; reverse sequence: CAGCCAGTAGATCCAGTGGAAG; amplicon of 135 pb), *β*-actin (forward sequence: 5′-AAGACCTCTATGCCAACAC-3′; reverse sequence: 5′-TGATCTTCATGGTGCTAGG-3′), and *β*2-microglobulin (forward sequence: 5′-ACTGGTCTTTCTACATCCTG-3′; reverse sequence: 5′-AGATGATTCAGAGCTCCATAG-3′). *β*-Actin and *β*2-microglobulin were used as reference genes. The reaction mixtures were kept for 45 min at 45°C, 2 min at 94°C, then cycled 35 times through a program of 30 s at 94°C, 1 min at 56°C, and 1 min at 72°C; finally, the reaction was incubated for an extra 7 min at 68°C. Normalized expression levels were calculated relative to control cells according to the 2^-ΔΔCT^ method.

### 2.5. Cell Transfection

B1647 cells were nucleofected with Cell Line Nucleofector™ Kit V (Amaxa Biosystems, Cologne, Germany) with Program T-019 following the manufacturer's instructions. SiRNA against Nox4 (sequence 5′-3′: CAACUCAUAUGGGACAAGAtt; antisense UCUUGUCCCAUAUGAGUUGtt) and scrambled were obtained from Ambion by Life Technologies (USA). RNA silencing was obtained with 300 nM siRNA. Subsequently, cells were immediately suspended in a complete medium and incubated in a humidified 37°C/5% CO_2_ incubator. After 24 h, cells were used for the experiments: evaluation of Nox4 expression by Western blot analysis and intracellular ROS level measurement.

### 2.6. Electrophoresis and Western Blot Analysis

After 24 h treatment with SFN (1, 5, or 10 *μ*M), B1647 cells (1 × 10^6^/mL) were washed with ice-cold PBS and lysed with RIPA buffer containing mammalian protease and phosphatase inhibitor mixtures. Protein concentration of the cleared lysates was determined by Bio-Rad DC™ protein assay. Proteins (10 *μ*g per lane) were electrophoretically separated on 4–20% SDS-PAGE Mini-Protean® TGX™ precast gels using a Mini-Protean II apparatus (Bio-Rad Laboratories) and transferred to Hybond-C nitrocellulose membrane. Nonspecific binding was avoided by incubating membranes in blocking buffer containing 5% (*w*/*v*) albumin in Tris-buffered saline (TBS)/Tween, then the nitrocellulose membranes were probed overnight at 4°C with primary antibodies (anti-AQP8, anti-Nox2, anti-phospho-VEGFR-2, anti-phospho-Akt, anti-Nox4, or anti-*β*-actin as internal normalizer). Nitrocellulose membranes were washed with TBS/Tween and incubated at room temperature for 1 h with horseradish peroxidase-conjugated secondary antibodies in TBS/Tween containing 5% nonfat dried milk or 5% albumin and successively washed with TBS/Tween. Chemiluminescence detection was performed using Clarity™ Western ECL Substrate. Bands were acquired with a CCD imager (ChemiDoc™ MP System, Bio-Rad Laboratories), and relative densitometric analysis were performed by using Image Lab analysis software (Bio-Rad Laboratories).

### 2.7. Immunofluorescence Confocal Microscopy

B1647 cells were treated with 10 *μ*M SFN for 24 h, loaded into cytospin chambers, and centrifuged at 450 rpm for 10 min. Cells were then fixed in formaldehyde 3.7% for 15 min, washed twice with PBS, blocked with PBS/BSA 1% (*w*/*v*) for 1 h, and incubated with mouse anti-AQP8 antibody for 1 h. Consecutively, cells were treated with fluorescent goat anti-Mouse Secondary Antibody, Alexa Fluor® Plus 488 conjugated for 1 h in the dark, nuclei were stained with DAPI, and coverslips were mounted on slides. Confocal imaging was acquired by a Nikon A1 confocal laser scanning microscope (Nikon Instruments, Japan).

### 2.8. Measurement of Intracellular ROS Level

B1647 cells were treated with 5 or 10 *μ*M SFN for 24 h and, when specified, with 1 *μ*M plumbagin for 30 min. To evaluate intracellular ROS level, 1 × 10^6^ cells/mL were washed twice in HBSS and incubated for 20 min with 5 *μ*M DCFH-DA at 37°C. DCFH-DA is a small, nonpolar, and nonfluorescent molecule that passes through the cell membrane into the cells by diffusion; in the cytosol, it is enzymatically deacetylated by intracellular esterases to a polar nonfluorescent compound, which is oxidised by intracellular ROS to the highly green fluorescent 2,7-dichlorofluorescein (DCF). DCF fluorescence was measured using a multiwell plate reader (Wallac Victor2, PerkinElmer). Excitation wavelength was 485 nm, and emission wavelength was 535 nm. Fluorescence values were reported as the percentage of intracellular ROS in respect to controls.

### 2.9. Immunoprecipitation

Control or SFN-treated B1647 cells (1 × 10^6^ cells/mL) were lysed as described above. Lysates containing equal protein amounts were incubated overnight with anti-AQP8 antibody. Then, samples were incubated with protein G-agarose for 1.5 h at 4°C and pelleted at 12,000 x *g* for 30 min. Pellets were washed 5 times with buffer (pH = 8) and centrifuged at 12,000 x *g* for 5 min. Samples were subjected to SDS-PAGE and Western blotting analysis with anti-Nox2 as described above. Bands were acquired with a CCD imager (ChemiDoc™ MP System, Bio-Rad Laboratories), and relative densitometric analysis were performed by using Image Lab analysis software (Bio-Rad Laboratories).

### 2.10. Statistical Analysis

Each experiment was performed at least three times, and all values are represented as means ± SD. One-way ANOVA was used to compare differences among groups followed by Bonferroni's test (Prism 5; GraphPad Software). Values of *p* < 0.05 were considered as statistically significant.

## 3. Results

**Figure 1:**
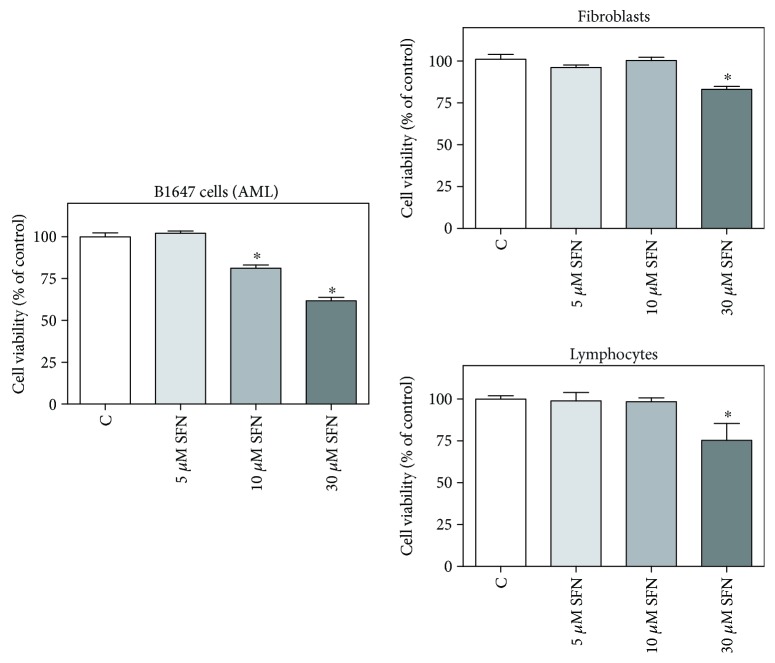
Effect of SFN on the viability of transformed and nontransformed human cells. B1647 cells, human lymphocytes or fibroblasts were incubated for 24 h with increasing SFN concentrations. Viability was evaluated by MTT test, as reported in Materials and Methods section. Results are expressed as means ± SD of three independent experiments. Statistical analysis was performed by Bonferroni multiple comparison test following one-way ANOVA. ^∗^*p* < 0.05, significantly different from control cells.

It has been reported that SFN is able to selectively exert cytotoxic effects in many human cancer cells without affecting normal cells [8]. On these bases, leukemia B1647 cells and human lymphocytes or fibroblasts, chosen as model of nontransformed cells, were incubated with increasing SFN concentrations, and cell viability was evaluated by MTT assay (Figure 1). Both 10 and 30 *μ*M SFN showed cytotoxic effects in B1647, as cell viability was significantly lower compared to control cells. In human lymphocytes and fibroblasts, 30 *μ*M SFN significantly reduced cell viability; meanwhile, 10 *μ*M SFN did not show any cytotoxic effect. Therefore, SFN concentrations below or at least the same as 10 *μ*M were used in the subsequent experiments.

**Figure 2:**
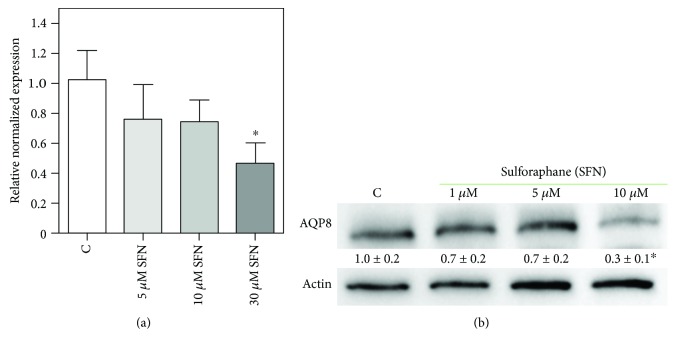
Effect of SFN on AQP8 expression in B1647 cell line. B1647 cells were incubated for 24 h with different SFN concentrations and (a) RNA was extracted from the cells and samples subjected to RT-PCR analysis using specific primers as described in Materials and Methods section. Normalized expression levels were calculated relative to control cells according to the 2^-∆∆Cq^ method; (b) proteins were extracted, separated by SDS-PAGE, transferred to nitrocellulose membrane, and immunoassayed using anti-AQP8 and anti-*β*-actin antibodies as reported in Materials and Methods section. Immunoblot is the representative of three independent experiments, and densitometric analysis, normalized to *β*-actin, is expressed as fold decrease with respect to control. ^∗^*p* < 0.05, significantly different from control cells.

To investigate the mechanism underpinning the observed cytotoxic effect of SFN in B1647 cells, we evaluated AQP8 expression after SFN treatment, as we hypothesized that SFN could impair the cellular redox status affecting H_2_O_2_ transport through AQP8 channel. In order to verify this hypothesis, B1647 cells were treated with different SFN concentrations for 24 h, and the expression of AQP8 was evaluated by RT-PCR (Figure 2(a)) and Western blot (Figure 2(b)) analyses.

Results show that AQP8 was significantly decreased both at transcriptional and protein level upon cell treatment with 10 *μ*M SFN, whereas 1 or 5 *μ*M SFN did not cause any significant change.

**Figure 3:**
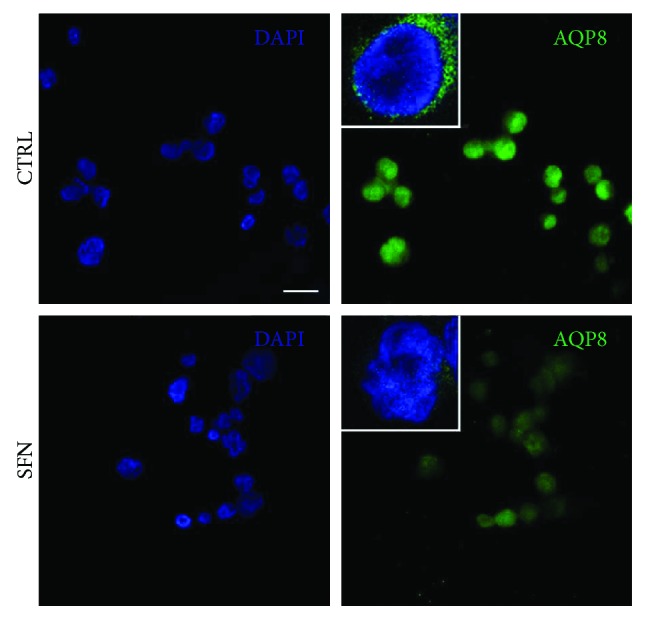
Effect of SFN on AQP8 content in plasma membrane of B1647 cell line. Representative confocal images of B1647 cells treated (SFN) or not (CTRL) with 10 *μ*M sulforaphane for 24 h and labelled with DAPI (blue) and anti-aquaporin 8, AQP8, (green). Cells were not permeabilized in order to exclude intracellular signals. Scale bar = 10 *μ*m. Triple magnification of representative superimposed 3 central slices is shown in white squares. Images were acquired by Nikon A1 confocal laser scanning microscope (Nikon Instruments, Japan). The results are the representative of two independent experiments.

To corroborate these findings, B1647 cells were treated with 10 *μ*M SFN, then AQP8 content in plasma membrane was evaluated using an immune-fluorescence technique and visualized through confocal microscopy (Figure 3).

As expected, SFN treatment strongly reduced green fluorescence in B1647 plasma membrane, confirming the ability of SFN to reduce AQP8 level, in agreement with RT-PCR and Western blot data.

**Figure 4:**
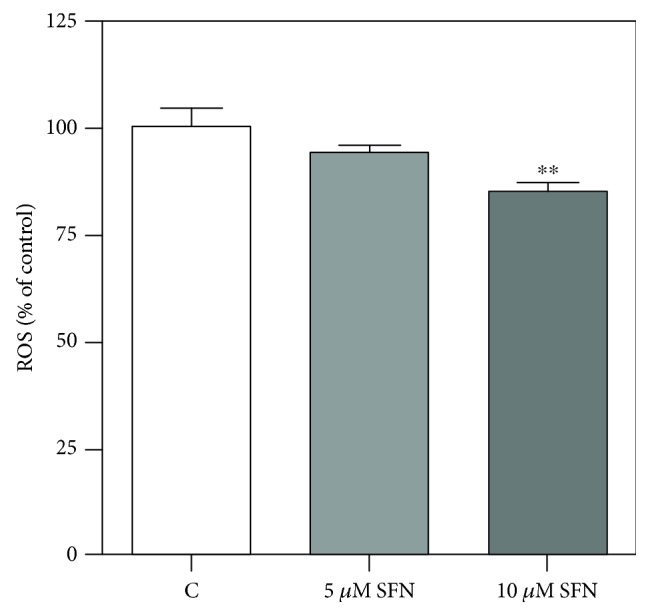
Effect of SFN on intracellular ROS level in B1647 cell line. B1647 cells were incubated for 24 h with different SFN concentrations. Intracellular ROS level was evaluated as DCF fluorescence as reported in Materials and Methods section. Data are expressed as % of control and represent means ± SD of at least three independent experiments. Data were analysed by one-way ANOVA followed by Bonferroni's test. ^∗∗^*p* < 0.01, significantly different from control cells.

Since SFN decreases AQP8 level, it is reasonable that a smaller amount of H_2_O_2_ is transported into the cell. To investigate this aspect, B1647 cells were incubated for 24 h with increasing SFN concentrations and then assayed for ROS level by using the fluorescent DCF probe. Results in Figure 4 show that only 10 *μ*M SFN treatment causes a significant decrease of ROS intracellular levels in respect to control cells, according to previous observations.

**Figure 5:**
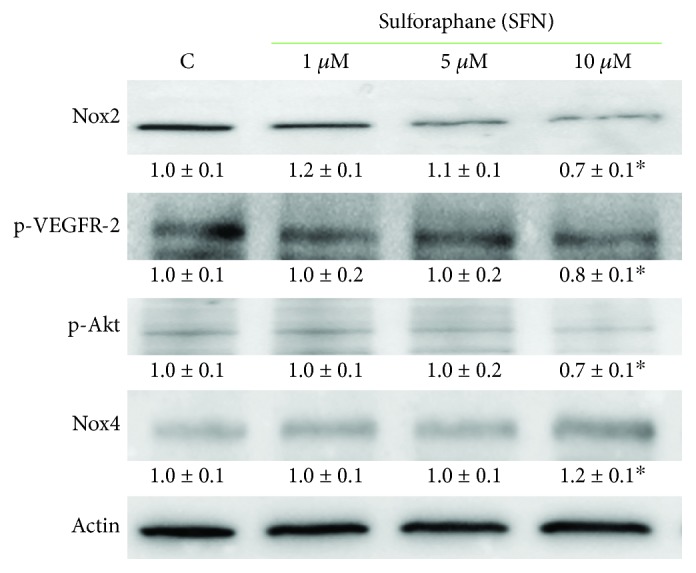
Effect of SFN on Nox2 and Nox4 expression and phosphorylation level of VEGFR-2 and Akt in B1647 cell line. B1647 cells were incubated for 24 h with different SFN concentrations. At the end of incubation, cells were lysed, and proteins were separated by SDS-PAGE, transferred to nitrocellulose membrane, and immunoassayed using specific antibodies as reported in Materials and Methods section. Immunoblots are the representative of three independent experiments, and densitometric analysis, normalized to *β*-actin, is expressed as fold decrease with respect to control. ^∗^*p* < 0.05, significantly different from control cells.

To better elucidate the mechanisms behind SFN ability to reduce ROS intracellular level, we investigated SFN influence on the sources of H_2_O_2_ present in B1647 cells. To this regard, we have previously demonstrated that the main sources of H_2_O_2_ in B1647 cell line are Nox2 and Nox4 isoforms [19]. Nox-derived ROS are involved in early signalling events, such as the auto-phosphorylation of VEGFR-2 leading to downstream events, including the maintenance of the active phosphorylated form of Akt [20]. Therefore, the possible effect of SFN on Nox isoforms expression and the phosphorylation level of VEGFR-2 and Akt were investigated in B1647 cell line (Figure 5).

**Figure 6:**
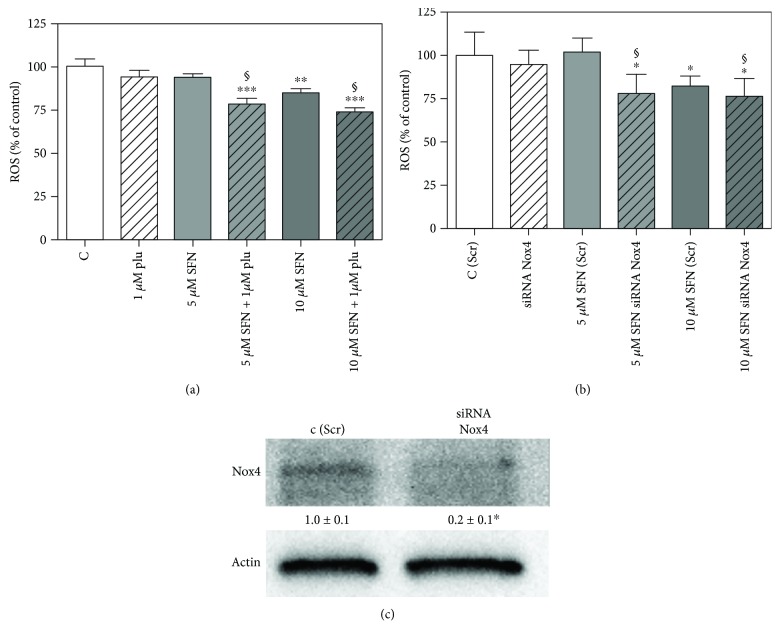
Effect of SFN on intracellular ROS level in B1647 cell line after Nox4 inhibition or silencing. (a) B1647 cells were incubated for 24 h with different SFN concentrations, then treated or not with 1 *μ*M plumbagin for 30 min. (b) B1647 cells were transfected with specific siRNA against Nox4 or a random RNA sequence (scrambled) as negative control, C (Scr). 24 h after transfection with siRNA, B1647 cells were incubated for 24 h with different SFN concentrations. Intracellular ROS level was then evaluated as DCF fluorescence as reported in Materials and Methods section. Data are expressed as % of control and represent means ± SD of three independent experiments. Data were analysed by one-way ANOVA followed by Bonferroni's test. ^∗∗∗^*p* < 0.001; ^∗∗^*p* < 0.01; ^∗^*p* < 0.05, significantly different from relative control cells. §*p* < 0.05, significantly different from the corresponding bars in the absence of plumbagin (a) or in Nox4-silenced cells (b). (c) B1647 cells were transfected by electroporation with siRNA against Nox4 or a random RNA sequence (scrambled) as negative control. Effect of RNA interference of Nox4 was confirmed by Western blot analysis with specific antibodies against Nox4.

Western blot analysis reveals that, when treated with 10 *μ*M SFN, B1647 cells express Nox2 to a lesser extent than controls and exhibit a diminished phosphorylation level of both VEGFR-2 and Akt. On the other hand, the amount of Nox4 was significantly increased. This result could explain the slight decrease in the level of intracellular ROS observed upon SFN treatment. To better appreciate SFN effect on Nox2, Nox4 isoform was inhibited by plumbagin, a Nox4 inhibitor [31], or by silencing with siRNA against Nox4.  Figure 6 shows the evaluation of intracellular ROS level in the presence of plumbagin (Figure 6(a)) or after specific Nox4 silencing (Figure 6(b)). Interestingly, SFN treatment led to a more pronounced reduction of intracellular ROS content when Nox4 was inhibited or silenced.

**Figure 7:**
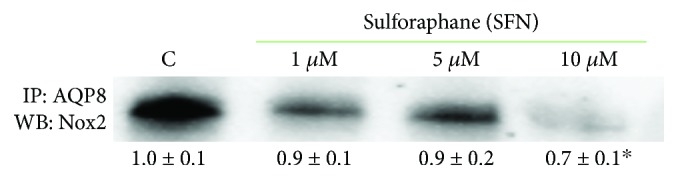
Effect of SFN on the interaction between AQP8 and Nox2 in B1647 cell line. B1647 cells were incubated for 24 h with different SFN concentrations. At the end of incubation, cells were subjected to immunoprecipitation with anti-AQP8. Proteins were then extracted, separated by SDS-PAGE, immunoblotted, and revealed for anti-Nox2 as described in Materials and Methods section. Immunoblot is the representative of three independent experiments, and densitometric analysis is expressed as fold decrease with respect to control. ^∗^*p* < 0.05, significantly different from control cells.

It has been recently reported that aquaporins can have protein interaction partners [31]; therefore, the possible interaction between AQP8 and Nox2, the main ROS source in B1647 cells, was investigated by immunoprecipitation technique. Results in Figure 7 show that Nox2 coprecipitates with AQP8, indicating a strong link between these two proteins. As expected, upon the 10 *μ*M SFN treatment, the band corresponding to Nox2 significantly lost intensity, according to the decreasing SFN effect on AQP8 and Nox2 expression. To corroborate this result, the “*vice-versa*” immunoprecipitation was performed, i.e., IP for Nox2 and WB for AQP8, and also, in this case, the coprecipitation of the two proteins was observed (not shown).

**Figure 8:**
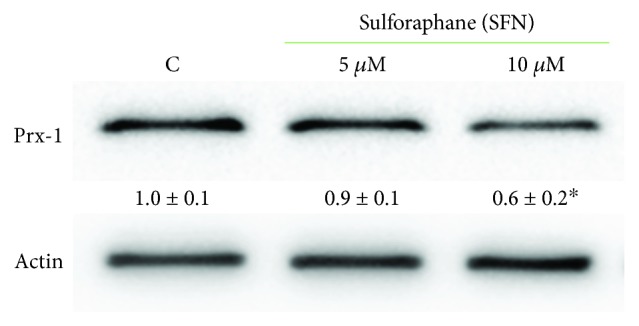
Effect of SFN on peroxiredoxin-1 (Prx-1) in B1647 cell line. B1647 cells were incubated with 5 or 10 *μ*M SFN for 24 h. At the end of incubation, cells were lysed, and proteins were separated by SDS-PAGE, immunoblotted, and revealed for anti-Prx-1 as reported in Materials and Methods section. Immunoblot is the representative of three independent experiments, and densitometric analysis, normalized to *β*-actin, is expressed as fold decrease with respect to control. ^∗^*p* < 0.05, significantly different from control cells.

Peroxiredoxins (Prxs) have catalytic cysteines exhibiting great susceptibility to oxidation by hydrogen peroxide [32]; therefore, they are important regulators of peroxide-dependent signalling pathways. Furthermore, Prxs have been found to be elevated in many human cancer cells and tissues, where they enhance the aggressive survival phenotype and confer increased resistance to chemo- and radio-therapy [33]. In order to ascertain whether SFN could affect Prx-1 expression, B1647 cells were incubated with SFN for 24 h, then subjected to Western blot analysis, as reported in Figure 8.

The results show that the cell treatment with 10 *μ*M SFN for 24 h significantly decreased Prx-1 expression.

## 4. Discussion

In a previous study carried out in B1647 cell line, we have demonstrated that AQP8 expression modulates the amplitude of the downstream VEGF signalling, which proceeds through the involvement of Nox-produced H_2_O_2_ as a second messenger [25]. Thus, this AQP isoform has gained an important role as a fine level regulator in the transduction of the redox signal. It has been reported that living cells can regulate the permeability of AQP8 to H_2_O_2_ and water through a temporary modification of functional cysteines, particularly during cell stress conditions [34, 35]. It seems of great interest the identification of molecules able to modulate the activity and/or expression of AQP8 isoform in order to influence the cellular response. In particular, the attention points toward the identification of natural products or food-derived molecules to be used as chemopreventive agents. On these premises, we investigated the potential effect of the isothiocyanate SFN on the modulation of AQP8 and Nox expression in the leukemic B1647 cell line. As a result of many *in vivo* and *in vitro* studies, it was stated that SFN is able to selectively exert cytotoxic effects in various human cancer cells, while having no cytotoxic effects, or being even cytoprotective in normal cells [5, 8]. The treatment of HL-60 cells with increasing concentrations of SFN (0–100 *μ*M) was reported to induce a dose-dependent decrease in cell viability, with a IC_50_ value determined as 49.5 *μ*M [36]; in pre-B ALL (acute lymphoblastic leukemia) and T-ALL cell lines, it was observed that SFN induce cytotoxicity at concentration ranging from 4 to 10 *μ*M in contrast to 90 *μ*M for nonleukemic controls [37]. These data evidence that high SFN concentrations are needed to exert cytotoxic effects on normal cells, whereas low concentrations provoke a selective effect on transformed cells. Our data show that 30 *μ*M SFN significantly reduced the viability of both leukemic and normal cells, while 10 *μ*M exerted a cytotoxic effect only in cancer cells. Therefore, SFN concentrations below or equal to 10 *μ*M were used in all the experiments. Interestingly, concentrations of similar order of magnitude can be really achieved in human plasma through dietary intake of cruciferous vegetables. Since it has been estimated that 40 g of fresh broccoli sprouts yield a transient serum level of SFN of about 2 *μ*mol/L [38], a serving of 200 g of broccoli can provide the desired SFN plasma level of about 10 *μ*mol/L.

As our results show that SFN is able to downregulate AQP8 expression, we can speculate that the lower level of intracellular ROS we measured by DCFH-DA is due to a smaller amount of H_2_O_2_ transported into the cell. DCFH-DA is not specific for H_2_O_2_ but reacts with all oxidants present in biological systems [39, 40]. However, in a previous paper of us, reporting data performed in the same leukemic cell line, i.e., B1647, we measured the intracellular ROS level with both DCFH-DA and PF1, a boronate dye more selective for H_2_O_2_ than DCFH-DA, obtaining similar results [24]. Furthermore, in the subsequent paper, we detected the intracellular thiol redox state of B1647 cells with a dimedone method, which is able to react with cysteine sulfenic acid, which is formed upon H_2_O_2_ action as a signalling molecule. We observed a linear correlation between the protein thiol redox state and the applied stimulus, i.e., H_2_O_2_ 10–100 *μ*M. This technique allowed us to demonstrate that the amplitude of intracellular cysteine oxidation is dependent on AQP8 expression level, which modulates the amount of H_2_O_2_ that is able to reach its intracellular targets [25].

The decrease of Nox2 expression observed in this study is, in part, counterbalanced by a significant increase in Nox4 expression. Indeed, we have previously demonstrated that B1647 cell line expresses Nox2 and the constitutively active Nox4, but not other isoforms [19]. It could be argued that cell undergoing SFN treatment might deploy mechanisms to counteract the limited H_2_O_2_ production (by Nox2) and transport (by AQP8) through the strengthening of Nox4 expression. Blocking Nox4 activity by the inhibitor plumbagin or knocking down this isoform by specific Nox4 silencing led to a more pronounced SFN effect on intracellular ROS content (Figure 6). In this condition, Nox2 remains the main ROS source in B1647 cells, and SFN effect can be better appreciated. Although it was reported that 10 *μ*M plumbagin greatly inhibited Nox4 activity in HEK293 and LN229 cells [41], in our conditions, this plumbagin concentration markedly decreased B1647 cell viability (data not shown), and therefore, we used 1 *μ*M plumbagin, according to Guida and coworkers [42].

By using a coimmunoprecipitation technique, we also demonstrated that Nox2 and AQP8 are linked to each other, confirming the existence of a Nox2-AQP8 axis in B1647 cell line. The evidence of the interaction between AQP8 and Nox2 supports the importance of these two partners in the redox signalling cascade. The activity of Nox2-AQP8 axis is also a determinant in B cell activation and differentiation [27]. Many isoforms of aquaporins have protein-protein interactions, specifically found for AQP0, AQP2, AQP4, and AQP5 [31]. The characteristics of these interaction partners are strikingly different, but they generally influence the translocation, trafficking, internalization, or phosphorylation of AQP isoforms. B1647 is a self-producing VEGF cell line, which is subjected to continuous VEGF signalling; therefore, the axis Nox2-AQP8 has a central role in modulating the downstream events supporting their viability and proliferation. This distinctive feature of these cells could explain the observed SFN-induced intracellular ROS decrease. Although other reports, obtained in different cell types, demonstrate a SFN-induced ROS increase [43–45], it has been shown that SFN inhibits VEGF expression [8, 9], which is strictly linked to Nox activation. Therefore, VEGF inhibition coupled with SFN-induced decrease of both Nox2 and AQP8 expression may contribute to the observed decrease in intracellular ROS level in B1647 cell line. Moreover, SFN is known to induce changes in the intracellular redox state, and, depending on its concentration, exposure time, or cell type, it may promote antioxidant or prooxidant response. From the data of the literature, it can be summarized that a predominantly antioxidant response has been reported at low SFN concentration, i.e., up to 5 *μ*M SFN for up to 24 h, which is close to our conditions, whereas higher SFN concentrations and long-lasting exposure periods produce a prooxidant effect [45, 46].

The ability of SFN to interfere with the redox signalling is confirmed also by its effect on VEGFR-2 and Akt phosphorylation status, which is significantly reduced in SFN-treated cells. The decreased amount of p-VEGFR-2 was an expected result, since ROS source involved in VEGFR-2 activation has been identified in Nox activity [20]. The smaller amount of p-Akt observed in SFN-treated cells indicates that this isothiocyanate exerts its action also on the downstream H_2_O_2_ targets, among which Akt represents a key enzyme controlling many hallmarks of cancer. Indeed, the active phosphorylated form of this enzyme plays a pivotal role in tumour cell survival, proliferation, and invasiveness [47].

Prxs are abundant thiol-dependent peroxidases highly efficient at reducing hydrogen peroxide, peroxynitrite, and other hydroperoxides [48]. Due to their high reactivity and abundance, Prxs will be the major targets of intracellular hydrogen peroxide [49] and, therefore, important regulators of peroxide-dependent signalling pathways [50]. Besides their antioxidant activity, recently, evidence indicates that Prxs have a significant influence on the development and progression of cancer. Prx-knock-out mice often exhibit increased carcinogenesis, whereas elevated Prx expression is commonly observed in human tumours [49]. In leukemia cells, Prxs display variable expression, suggesting difference in functional significance depending on the cellular context [51]. In particular, a proteomic analysis has demonstrated that Prxs are significantly increased in almost all acute myeloid leukemia (AML) subtypes; thus, they were proposed as potential targets for AML patients [52]. Our results show that B1647 cell line expresses Prx-1, which is significantly reduced upon SFN treatment, indicating an additional protective role of this isothiocyanate against malignancy. However, further studies are needed to elucidate a definitive role for Prx family in leukemia.

## 5. Conclusions

The data reported here show that SFN downregulates AQP8 and Nox2 expression in B1647 cell line, limiting both H_2_O_2_ production and entry into the cells. Consequently, the amount of hydrogen peroxide able to reach its intracellular targets is decreased, and leukemia cell viability significantly reduced. Indeed, by decreasing the effect of Nox2-AQP8 axis, SFN causes profound effects on the transduction of the redox signalling and, consequently, on cell survival and proliferation, opening the way to unforeseen opportunities in the fighting of acute myeloid leukemia. Of note, SFN concentrations able to trigger these effects are comparable to plasma concentrations measured after cruciferous vegetables dietary intake.

## Data Availability

The data used to support the findings of this study are available from the corresponding author upon request.

## Abbreviations

SFN: Sulforaphane
AQP8: Aquaporin-8
VEGF: Vascular endothelial growth factor
VEGFR-2: Vascular endothelial growth factor receptor-2
Nox: NAD(P)H oxidase
Prx: Peroxiredoxin.
